# The Impact of N-Acetylcysteine on Autologous Fat Graft: First-in-Human Pilot Study

**DOI:** 10.1007/s00266-020-01633-1

**Published:** 2020-03-27

**Authors:** Piotr Pietruski, Wiktor Paskal, Łukasz Paluch, Adriana M. Paskal, Żaneta Nitek, Paweł Włodarski, Jerzy Walecki, Bartłomiej Noszczyk

**Affiliations:** 1 Department of Plastic and Reconstructive Surgery, Medical Centre of Postgraduate Education, Prof. W. Orlowski Memorial Hospital, Warsaw, Poland; 2 Laboratory of Center for Preclinical Research, Department of Methodology, Medical University of Warsaw, Stefana Banacha 1B, 02-097 Warsaw, Mazowieckie Poland; 3 Department of Radiology, Medical Centre of Postgraduate Education, Gruca Orthopaedic and Trauma Teaching Hospital, Otwock, Poland

**Keywords:** Acetylcysteine, Autologous fat graft, Breast, Lipofilling, Oxidative stress

## Abstract

**Background:**

Our goal was to determine whether N-acetylcysteine (NAC) administered to the tumescent solution can reduce oxidative stress and increase autologous fat graft (AFG) viability.

**Methods:**

The study included 15 women with a mean age of 31.8 years (range 23–39 years) who underwent breast asymmetry correction with AFG harvested from both thighs. One thigh was infiltrated with a standard tumescent fluid (control graft) and other with a NAC-enriched tumescent fluid (NAC-treated graft). Each participant had breast MRI imaging before and 6 months after the procedure. Also, adipose tissue samples from each graft were subjected to biochemical analysis, flow cytometric assay and qRT-PCR to determine the markers of oxidative stress, angiogenesis and adipogenesis.

**Results:**

Concentration and activity of superoxide dismutase in the NAC-treated grafts turned out to be significantly higher than in the control grafts, in both fresh (*p* = 0.041 and *p* = 0.023, respectively) and frozen samples (*p* = 0.004 and *p* = 0.003, respectively). The level of nitric oxide in frozen samples from the control grafts was significantly higher than in the NAC-treated grafts (*p* = 0.009). iNOS was the only qRT-PCR target showing significant intergroup differences, with higher transcription levels observed in the control grafts (*p* = 0.027). Breast volumetric analysis demonstrated that the NAC-treated group had a 12.19% lower resorption rate than the control group, although it was found to be statistically insignificant (*p* = 0.149). No postoperative complications were observed during a 6-month follow-up.

**Conclusions:**

Some results of this study are promising. Further studies on larger groups are needed to determine NAC impact on AFG.

**Level of Evidence IV:**

This journal requires that authors assign a level of evidence to each article. For a full description of these Evidence-Based Medicine ratings, please refer to the Table of Contents or the online Instructions to Authors www.springer.com/00266.

**Trial registry name:**

The Impact of N-Acetylcysteine on Volumetric Retention of Autologous Fat Graft for Breast Asymmetry Correction.

**Registration identification number:**

NCT03197103.

**URL for the registry:**

https://clinicaltrials.gov/ct2/show/NCT03197103?term=acetylcysteine&amp;rank=6

**Electronic supplementary material:**

The online version of this article (10.1007/s00266-020-01633-1) contains supplementary material, which is available to authorized users.

## Introduction

Over the recent 20 years, autologous fat grafting (AFG) has become a widely recognized and applied technique in both aesthetic and reconstructive surgeries [1–15]. The widespread use of AFG is associated with the simplicity of the surgical procedure, quick recovery and low morbidity, as well as with not infrequently spectacular immediate postoperative results [1, 2, 10, 12].

A primary argument of AFG critics is unpredictable long-term viability of the transplanted fat. According to various reports, the graft resorption rate may vary from 10% up to 90% [1, 10, 16–21]. Theoretically, adherence to the principles of fat grafting should be reflected by constant, low retention rates [22]. Also, this clinical issue could be partially solved with additional grafting procedures or overcorrection. However, the first solution is associated with an increase in overall treatment cost. Moreover, in some cases, the second harvesting procedure may not be possible due to too small volume of adipose tissue at the donor site. Many previous studies demonstrated that a compensation of expected volume retention via overcorrection may result in unfavorable graft-to-recipient interface [23]. This is associated with the increased risk of oil cyst and fibrotic scar tissue formation and fat necrosis, which eventually leads to an even greater resorption rate and unfavorable esthetic outcomes [7, 23–26].

No standardized and widely recognized protocol or guideline to optimize AFG has been published thus far [27]. Generally, each fat grafting procedure consists of four stages: graft harvesting, also referred to as procurement, cell processing, transplantation and management of the recipient site [10, 28, 29]. Theoretically, each of these stages might influence the final outcome, exerting an effect on volume retention. The resorption is postulated to result from a cascade of mechanical damage, ischemic/hypoxic injury and oxidative stress. Surprisingly, however, direct prevention of oxidative stress at the time of fat graft harvesting has been a subject of only a few previous studies [30, 31].

N-acetylcysteine (NAC) not only acts as a free radical scavenger but is also capable of maintaining the intracellular status of glutathione, a key endogenous antioxidant [32]. NAC has many established clinical applications, among them prevention of contrast-induced nephrotoxicity and acetaminophen hepatotoxicity, and some other potential applications are a subject of ongoing research [31]. Unquestioned advantages of NAC include its low cost and favorable safety profile, with only few contraindications, such as allergic reaction, asthma and active peptic ulcer disease [32, 33].

The aim of this pilot study was to verify the ability of NAC to inhibit the inflammatory cascades triggered by soft tissue manipulation, its possible impact on the fat graft retention rate and potential complications resulting from its application.

## Methods

### Patients

The protocol of the study was approved by the institutional review board, and written informed consent was sought from all participants. The study included 15 female volunteers willing to undergo at least a two-stage AFG procedure to correct their breast asymmetry. Inclusion and exclusion criteria of the study are listed in Table 1. The age of the study participants ranged from 23 to 39 years (mean 31.8 years), and their mean body mass index (BMI) amounted to 22.41 kg/m^2^.

**Table 1:** Study eligibility criteria

Inclusion criteria	Exclusion criteria
Female Age 18–40 years BMI > 20 kg/m2 Hypoplastic, asymmetric breasts ASA I according to the ASAPS classification system	A history of allergic reaction to NAC Asthma Peptic ulcer disease Contraindications to MRI Breastfeeding, pregnancy or pregnancy planned within a year A history of breast surgery or radiotherapy A history of hip/thigh surgery Illness or general state of health precluding general anesthesia

### MRI Imaging

All participants underwent breast MRI before the AFG procedure. Each MRI study was performed with the patient in the prone position, in a dedicated phased-array breast coil using a 1.5-T imager (Optima MR360, GE Medical Systems, Milwaukee, WI). The imaging protocol consisted of turbo short TI inversion recovery sequence in the axial plane (TR/TE 3470 ms/40 ms), an axial T1 (TR/TE 700 ms/ 1 ms), an axial T2 (TR/TE 4548 ms/84.9 ms), an axial T1 fs Vibrant (TR/TE 5.7 ms, 2.7 ms), a sagittal T2 (TR/TE 4834 ms, 104 ms) and a fat-saturated axial T2 (TR/TE 4831 ms, 84.9 ms). Control MRI of the breasts was performed according to the same imaging protocol six months after the AFG procedure.

### Autologous Fat Graft Acquisition and Administration

Each patient received a 2 g intravenous dose of cefazolinum thirty minutes before the surgery. All fat graft acquisition procedures were carried out by the same operator to avoid inter-operator variability. The operator was blinded to the assignment of tumescent solutions. Each participant of the study underwent suction-assisted tumescent liposuction of the anterior and inner thigh under general anesthesia. Two types of tumescent solutions were used: Klein solution (1 l of saline with 50 ml of 1% lidocaine, 1 ml of 1:1000 epinephrine) administered to one thigh (control grafts) and Klein solution enriched with NAC, administered to another thigh (NAC-treated grafts).

For enriching tumescent solution with NAC, we used 300 mg/3 ml acetylcysteine intravenous solution (Sandoz, Holzkirchen, Germany). Per each liter of the solution, 600 mg of NAC was added (0.06% NAC solution). This concentration allowed us not to exceed 1200 mg of NAC, which is considered a safe-to-use daily dosage recommended, for example, in contrast-induced nephropathy. Since there are no pharmacokinetic data available for subcutaneous administration of NAC, we were obligated by our institutional bioethics committee not to exceed this dosage in our pilot study [34]. Results of yet an unpublished study (PhD thesis of the second author) conducted on a rat animal model indicate that subcutaneous and intradermal administration of NAC solution of a concentration of 0.03% and higher, lowers the oxidative stress injury significantly and enhances wound healing. This finding seems to correspond well to the conclusions of the other animal studies, showing that a similar NAC dosage reduces oxidative stress and inflammation as well as improves wound healing along with neovascularization and nerve regeneration [35–37]. Those facts allowed us to presume that using a NAC solution of concentration 0.06% would allow reducing the oxidative stress significantly in female volunteers.

Consequently, the thighs of each patient served as a source of both experimental and control grafts. The thigh administered a given tumescent solution was selected randomly, by coin tossing. The infiltration was carried out with a 6-hole infiltration cannula (Marina Medical Inc., Davie, FL). Both thighs of each patient were infiltrated with the same volume of 1750 ml of either solution. The suction-assisted liposuction (240 mmHg vacuum) with a Khouri Harvester Cannula (Marina Medical Inc., Davie, FL) was carried out 30 min later. Each lipoaspirate (approximately 1.5 l) was collected to a separate canister whereby it underwent a sedimentation and static decantation to isolate the adipose layer. Immediately after the isolation, 65 ml of harvested fat was transported to the laboratory for analysis.

**Fig. 1:**
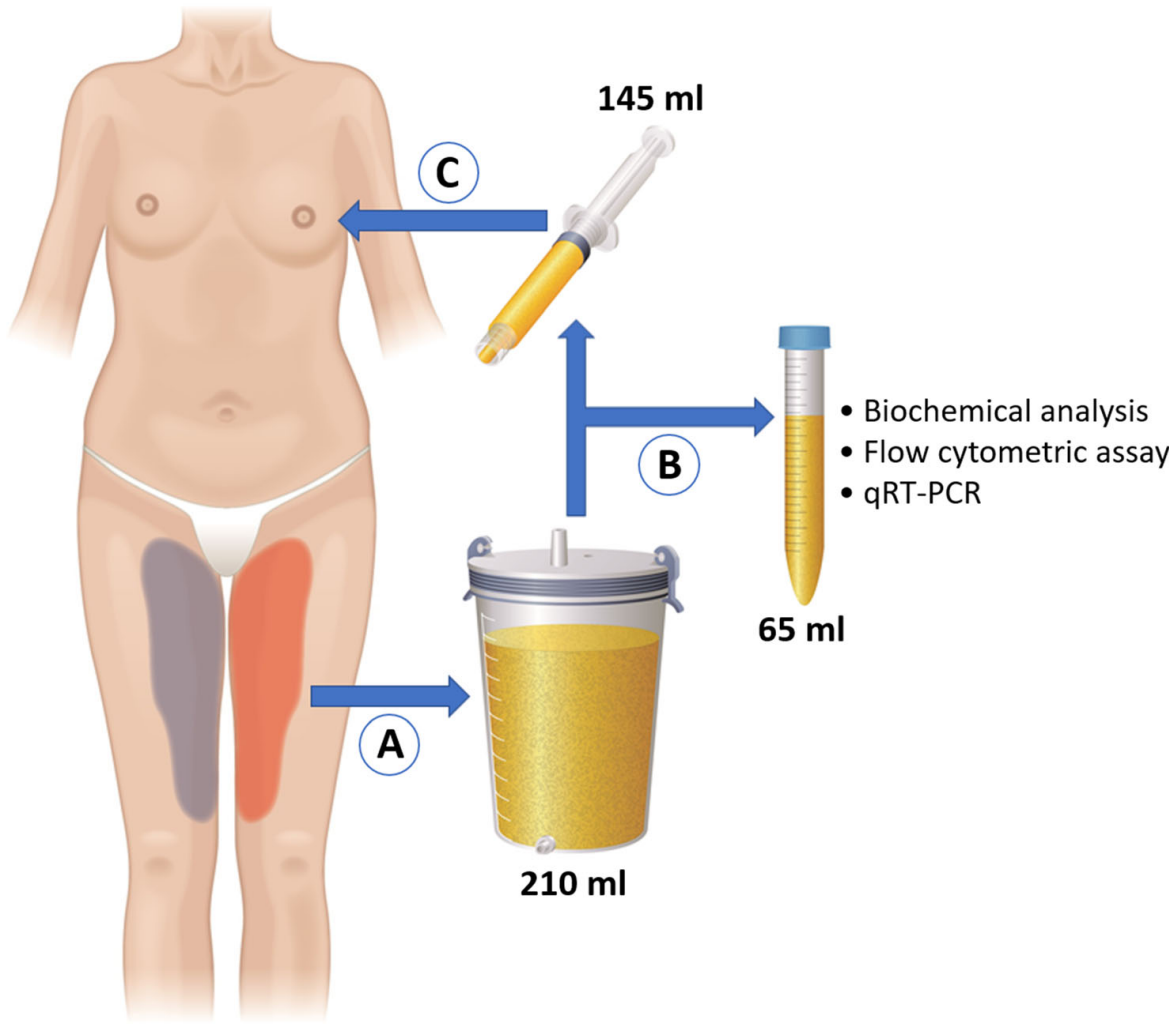
Autologous fat graft acquisition and usage framework. **a** Infiltration of each thigh with either Klein control tumescent solution or solution enriched with NAC. Using the suction-assisted liposuction technique, lipoaspirate from each thigh was collected to a separate canister. **b** After sedimentation and static decantation, a total of 65 ml of AFG was sent for biochemical, flow cytometric and genetic analyses. **c** A total of 145 ml of NAC-enriched AFG was implanted to one breast, while the same volume of the control AFG was injected to the other breast

Each of two breasts, selected randomly by coin tossing, was injected with 145 ml of either control or NAC-treated AFG. The fat was injected in small quantities (2-ml syringe) into multiple microtunnels with a cannula (COL-119CV, Mentor Medical Systems, Netherlands), through twelve entry points located at the breast margins. To minimize inter-operator variability, all breast augmentation procedures were carried out by the same operator blinded to the type of injected AFG (Fig. 1). In the postoperative period, patients were given paracetamol as an analgesic agent.

### Biochemical Evaluation of Oxidative Stress

The severity of oxidative stress was determined based on the levels of reactive oxygen species (ROS) and nitric oxide (NO), along with the concentration and activity of superoxide dismutase (SOD). Analysis of each assay was performed according to the same protocol on both fresh (within 120 min after harvest) and frozen (stored in -20 °C) samples of adipose tissue.

For ROS determination, a H2-DCF kit (Thermo Fisher Scientific, Waltham, MA) was used. The prepared and stained adipose tissue lysates were analyzed with a microplate fluorescence reader (FLUOstar Omega, BMG LABTECH, Germany). Each sample was measured 3 times at 520 nm with an excitation wavelength of 485 nm.

Samples for SOD and NO assays were discarded from wash buffer of 500 µl, and adipose tissue was transferred into a 2-ml tube and mixed with 500 µl ice-cold lysis buffer (0.1 M Tris/HCl, pH 7.4 containing 0.25 M sucrose, 5 mM ß-ME, 0.1 mg/ml PMSF). Then, manual homogenization was done with an Omni Tissue Homogenizer TH220 (Omni International, Kennesaw, GA) for 30 s. Homogenates were centrifuged at 15,000 g for 30 min at 4 °C. Finally, a clear supernatant was taken for further analyses, and 50 µl of Triton X-100 was added to each milliliter of the supernatant.

NO concentration in an adipose tissue sample was assessed using Griess reagent (Sigma-Aldrich, Saint Louis, MO). To determine a standard curve, the following concentrations of sodium nitrite were used (Sigma-Aldrich, Saint Louis, MO): 100 μM, 50 μM, 25 μM, 12.5 μM, 6.25 μM, 3.13 μM, 1.56 μM, 0 μM. One hundred microliters of supernatants (triplicates) after adipose tissue preparation was mixed 1:1 with Griess reagent. The absorbance at 540 nm was read after 15 min at the microplate fluorescence reader (FLUOstar Omega, BMG LABTECH, Germany).

SOD activity and concentration were determined with an SOD Assay Kit-WST (Sigma-Aldrich, Saint Louis, MO). The absorbance of each sample was read at 450 nm with a microplate fluorescence reader (FLUOstar Omega, BMG LABTECH, Germany). For determination of absolute SOD concentration, standard SOD solutions (Sigma-Aldrich, Saint Louis, MO) were prepared (range from 0.001 to 200 U/ml). SOD activity was calculated with a manufacturer formula, including negative and positive controls.

### Cytometric Analysis

The adipose layer of lipoaspirate was washed with sterile PBS three times, each time the infranatant has been discarded. The extracellular matrix was digested with 0.1% collagenase type Ia (Sigma-Aldrich, Saint Louis, MO) and incubated for 1 h at 37 °C. After enzymatic digestion, the infranatant containing the vascular fraction survival (SVF) was pipetted into sterile 50-ml centrifuge tubes. Equal to infranatant volumes of control medium were added and incubated at room temperature for 5 min. Then, the suspension was centrifuged at 1200 × g for 10 min. After removal of the supernatant, the SVF pellet was suspended in 10 ml of sterile 160 mM NH_4_Cl for red blood cell lysis and incubated at room temperature for 10 min, followed by 1200 × g /10 min centrifugation [38].

Cell viability was assessed by staining with 0.4% trypan blue and counting in a Bürker chamber (Sigma-Aldrich, Saint Louis, MO) and verified during flow cytometry. For more precise estimation of SVF viability, Hoechst (H) and iodium propide (PI) (Thermo Fisher Scientific, Waltham, WI) staining was implemented. Around 200,000 stained cells were analyzed with a CytoFLEX flow cytometer (Beckman Coulter, Indianapolis, IN). Three populations of cells were distinguished: live cells with low H and PI fluorescence, apoptotic cells with high H and low PI fluorescence and dead cells with only high PI fluorescence. Results were analyzed in FlowJo (FlowJo LLC, Ashland, OR).

### Quantitative Real-Time Reverse Transcription Polymerase Chain Reaction

Analysis of gene expression related to oxidative stress response and viability of adipose cells was analyzed with quantitative real-time reverse transcription polymerase chain reaction (qRT-PCR). Adipose tissue harvested in RNALater® (Thermo Fisher Scientific, Waltham, WI) was transferred into a  − 80 °C freezer. All samples were thawed and homogenized with Omni TH (Omni International, Kennesaw, GA). Total RNA was isolated with a silica column-based kit GeneMATRIX Universal (EurX, Gdansk, Poland). One hundred nanograms of total RNA from each sample was used for reverse transcription (GoScript, Promega Company, Madison, WI). qRT-PCR was performed with Power SYBR Green (Thermo Fisher Scientific, Waltham, WI) and gene-specific pair of primers for precise determination of genes’ expression (Table 2). GAPDH and ACTB were endogenous reference gene expression controls. qRT-PCR was conducted in 384-well plates. Each reaction was performed in triplicate in a QuantStudio Real-Time PCR System (Thermo Fisher Scientific, Waltham, WI).

**Table 2:** Summary of the analyzed genes

Determinant	Gene
Oxidative stress	Superperoxide dismutase (hsSOD) Heme oxygenase 1 (HO-1) Inducible NO synthase (iNOS) Glutathione peroxidase 3 (GPX-3) Catalase (hsCAT)
Adipogenesis	Peroxisome proliferator-activated receptors (PPAR-γ) CCAAT/enhancer-binding proteins (C/EBP β)
Angiogenesis/vascularization	Vascular endothelial growth factor (VEGF) Angiopoietin-2 (ANG-2)
Gene expression control	Glutathione peroxidase 3 (GPX-3) Catalase (hsCAT)

### MRI Analysis

Two blinded radiologists independently analyzed pre- and postoperative MRI data of 14 participants using 3D Slicer software (https://www.slicer.org) [39]. Each breast volume was measured within the following borders: midsternal line as the medial border, lateral thoracic artery defining the lateral chest wall and border, the internal fascia of both pectoral muscles as the inferior and the skin layer as the superior borders. Knowing the difference between the pre- and postoperative breast volumes and the volume of AFG used for breast augmentation, we were able to estimate the AFG resorption rate. In addition, MRI images were evaluated for the presence of fat necrosis, calcifications, scarring and oil cysts.

### Postoperative Complications

Each patient was followed up for potential systemic and local postoperative complications. Inspection of both donor site and augmented breasts was carried out at discharge from the hospital, as well as two weeks, three and six months after the procedure. The list of potential complications included hematoma, seroma, infection, skin necrosis, dermatitis or cellulitis, pain at the liposuctioned site exceeding three points on visual analogue scale and discomfort persisting for more than three weeks.

### Statistical Analysis

Normal distribution of the study variables was verified with a Kolmogorov–Smirnov test. Intergroup comparisons were made with a Mann–Whitney *U* test. For the AFG volumetric changes comparison, a Wilcoxon test was used. The inter-rater reliability, expressed as interclass correlation (ICC) for the radiological MRI examination, was analyzed with the Pearson correlation test. In addition, a *t* test was conducted for not related samples. All calculations were conducted with Statistica 10 (StatSoft, Tulsa, OK, USA), with the threshold of statistical significance set at *p* < 0.05.

## Results

**Fig. 2:**
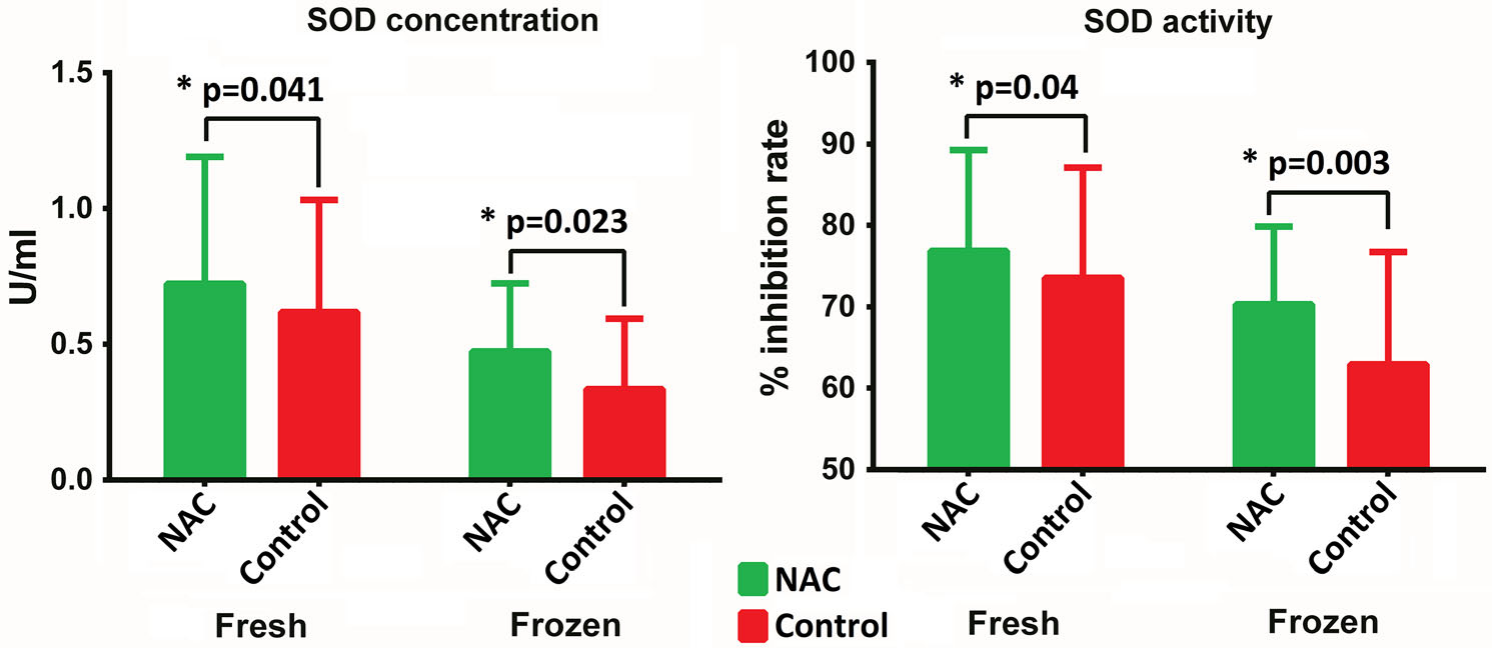
Intergroup differences in the concentrations and activity of SOD in fresh and frozen AFGs

**Fig. 3:**
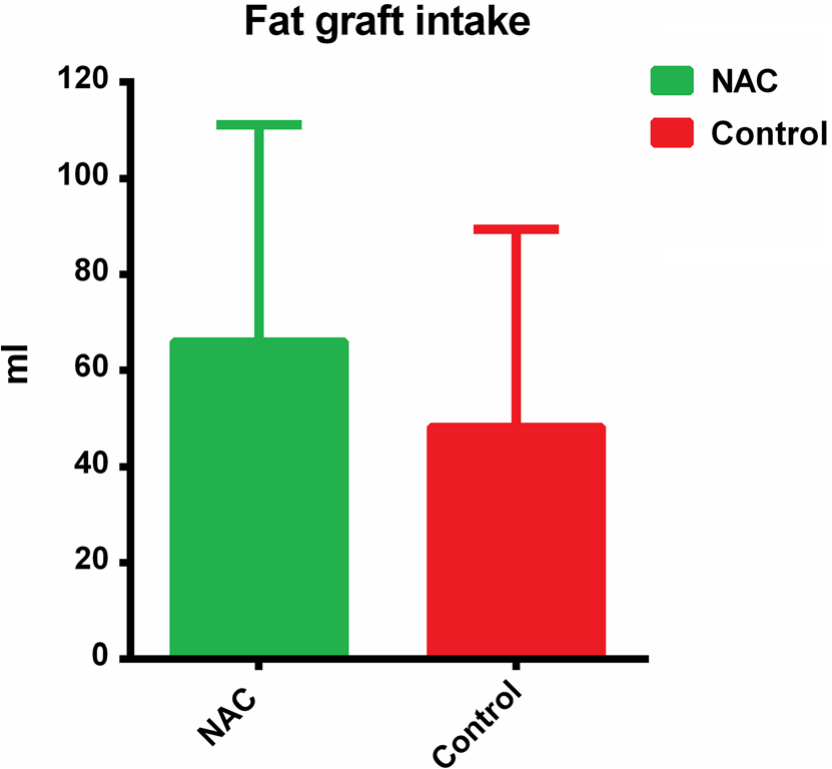
Intergroup differences in the levels of NO in fresh and frozen AFGs

**Fig. 4:**
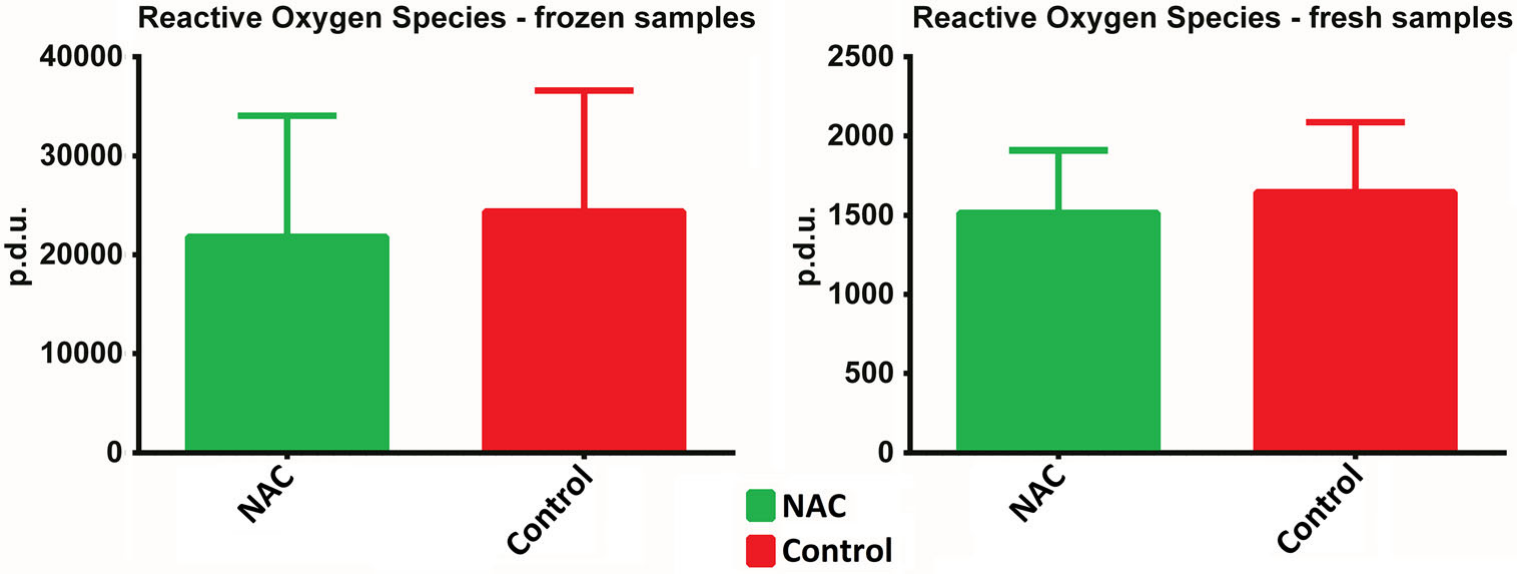
Intergroup differences in the levels of ROS in fresh and frozen AFGs

Detailed results of biochemical analysis, flow cytometry and qRT-PCR are presented in Supplementary data (Supplementary Table 1). Statistical analysis demonstrated that concentration and activity of SOD in the NAC-treated grafts, both fresh (*p* = 0.041 and *p* = 0.023, respectively) and frozen samples (*p* = 0.04 and *p* = 0.003, respectively) were significantly higher than in the control grafts (Fig. 2). The level of NO in frozen control grafts was significantly higher than in the NAC-treated grafts (*p* = 0.009) (Fig. 3). Interestingly, no statistically significant intergroup difference was found in ROS levels in either fresh or frozen samples (Fig. 4).

**Fig. 5:**
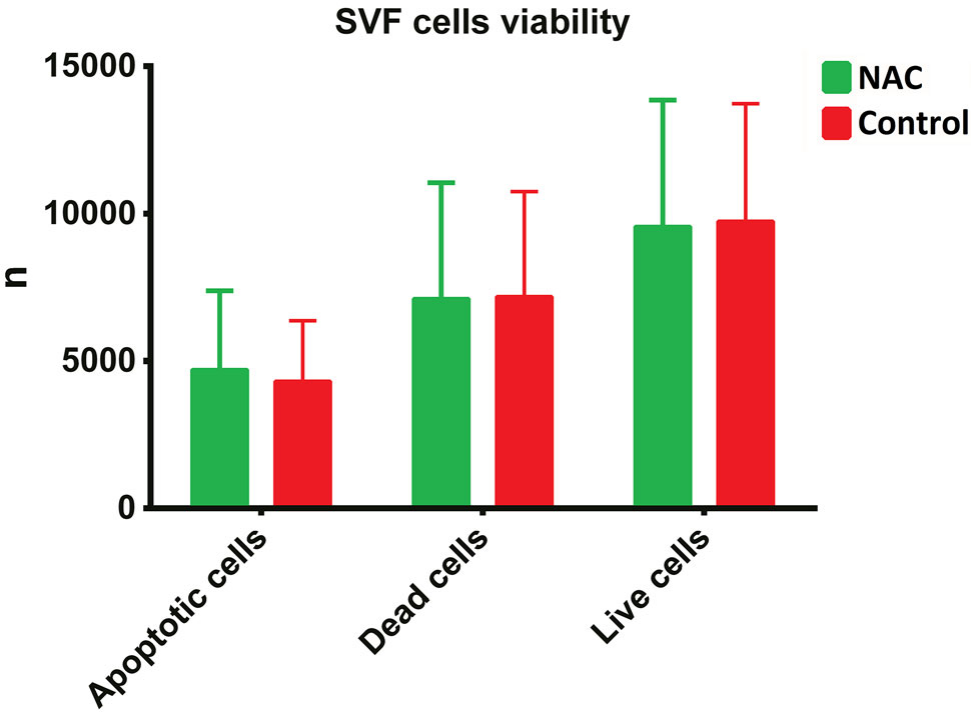
Comparison of flow cytometry results

**Fig. 6:**
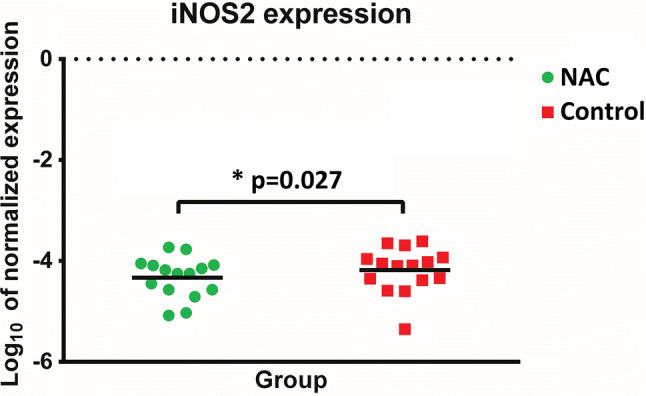
Comparison of iNOS gene expressions

Flow cytometric analysis did not demonstrate statistically significant differences in survival of SVF cells (Fig. 5). The only qRT-PCR target showing significant intergroup differences was iNOS (encoding inducible NO synthase), with higher transcription levels observed in the control grafts (*p* = 0.027) (Fig. 6).

**Fig. 7:**
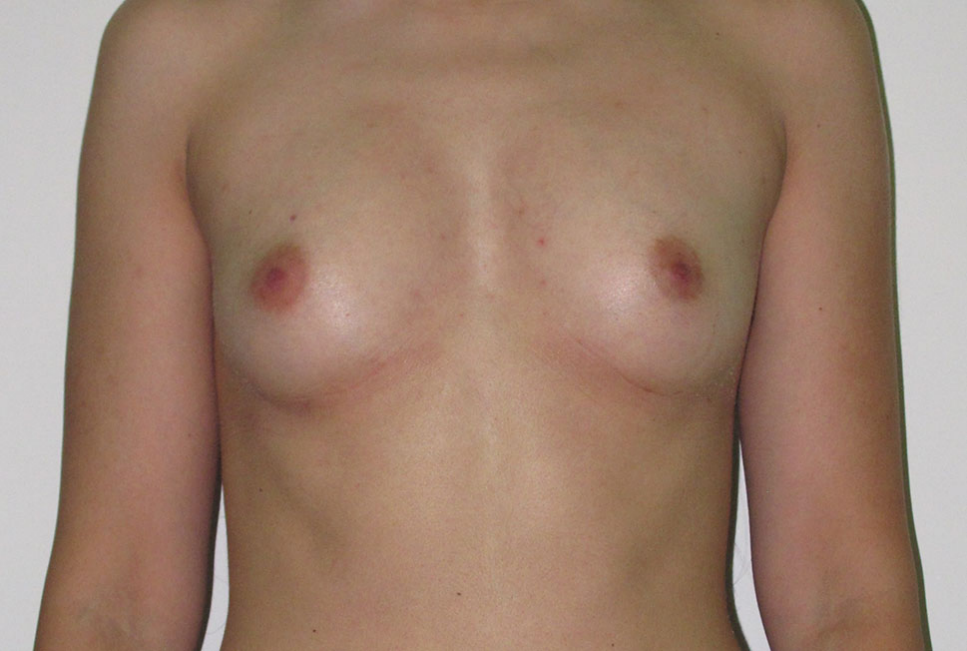
Breast appearances after 6 months. No noticeable size difference can be observed

One patient was excluded from this part of the study because of her migration and inability to refer for postoperative MRI at the specified time. The two radiologists did not differ significantly in terms of their volumetric estimates (ICC = 0.999 with *p* < 0.001). *T* test results confirmed this finding (*p* = 0.345). A significant decrease in AFG volumes was observed in both the NAC (*p* = 0.001) and control groups (*p* = 0.001). The fat intakes in the NAC and control groups were estimated at 66.11 ± 45 ml and 48.43 ± 40.99 ml, respectively, out of the initial 145 ml. Although the AFG survivability in the NAC group was 12.19% higher than in the control group, the between-group difference was not statistically significant (*p* = 0.149). No fat necrosis lesions, calcifications or large fibrotic scar formation was found. A single oil cyst was detected in three patients. Two cysts were localized in the control breast and one within breast augmented with NAC-enhanced graft. During the physical examination, no visible size differences between breasts were observed by the examiner in either patient (Fig. 7).

Neither local nor systemic postoperative complications were observed in the study participants during a 6-month follow-up.

## Discussion

For over 20 years, since the AFG technique was first presented by Coleman, a number of studies analyzed perspectives for the improvement of adipocyte survival [1]. The studies have been dealing with virtually all aspects of the AFG procedure, from the shape and size of the cannula to the effects of graft processing and transplantation technique. Unfortunately, most of the experimental adjunctive techniques are still at preclinical stages, and the results of some studies cannot be easily translated into routine clinical practice, are difficult to compare or do not provide reliable (level I or level II) evidence.

According to some authors, the initial stage of the AFG procedure, i.e., harvesting, is critical for subsequent graft survival, and every effort should be made to minimize soft tissue injury during this phase [19]. However, complete elimination of mechanical and ischemic stress is impossible, which leads to activation of inflammatory cascades, resultant adipocyte injury and adipose tissue resorption [12, 31, 40–44]. Lethal adipocyte injury is a consequence of oxidative stress caused by ROS, such as hydrogen peroxide and superoxide radicals, during the initial stage of the inflammation [40, 44].

While most studies aimed at the improvement of AFG outcomes have been centering around the processing and management of the recipient site, our project was one of few focusing at intervention at the earliest stage of the procedure. We hypothesized that infiltration of the donor site with a solution enriched with NAC, a well-known antioxidant agent, before its exposure to inflammatory triggers, i.e., tissue injury, might counteract or reduce oxidative stress. This, in turn, should result in lesser adipocyte injury and eventually reduce the graft resorption rate.

SOD is a key antioxidant enzyme protecting cells against the toxicity of ROS, including NO. Biochemical analysis demonstrated that both fresh and frozen AFG samples from the NAC-treated grafts had significantly higher concentrations (*p* = 0.041 and *p* = 0.004) and activities (*p* = 0.023 and *p* = 0.003) of SOD than the control grafts (*p* = 0.041 and *p* = 0.004). These findings imply that NAC might protect the grafts against oxidative stress. However, we did not observe statistically significant differences in the levels of ROS in NAC-treated and control grafts, as well as in the concentrations of NO in the fresh samples. Interestingly, however, the levels of NO in frozen NAC-treated grafts turned out to be significantly lower than in the control grafts (*p* = 0.009). This implies that NAC might improve preservability of the frozen adipose tissue. This property of NAC might find an application during in vitro studies, for example, for a culture of harvested adipose cells.

qRT-PCR demonstrated that the transcription level of the iNOS gene in the control grafts was significantly higher than in the NAC-treated grafts. iNOS encodes inducible NO synthase, an enzyme responsible for the synthesis of NO. Our observation corresponds well with the elevated concentrations of NO found in the control grafts deprived of potential protective effects of NAC. However, we did not find statistically significant differences between NAC-treated and control grafts in the expressions of other analyzed genetic markers of oxidative stress, angiogenesis and adipogenesis. The lack of significant differences in the expressions of VEGF and ANG-2 genes, both involved in the process of angiogenesis, seems to be consistent with the results of a previous study in which NAC exerted no effect on the vascularity of fat graft [30].

Flow cytometry did not show significant differences in the proportion of live, dead and apoptotic cells in both groups. These findings suggest that NAC had no beneficial effect on the early survival of cells obtained by lipoaspiration. However, our results imply also that the enrichment of tumescent solution with NAC did not exert an unfavorable effect on the harvested cells and hence may be considered a safe procedure.

Comparative volumetric analysis of the AFGs six months after their implantation to the breasts demonstrated that the resorption rate in the NAC group was approximately 12% lower than in the control group. At this stage of the study, we cannot exclude that the lack of a statistically significant difference in the resorption rates of NAC-treated and control grafts was associated with a relatively small size of the study group.

Our study has several limitations. First, due to the preliminary character of the study, the number of participating patients was relatively small. Importantly, none of the participants presented with any local or systemic postoperative complications during a 6-month follow-up period. This implies that the infiltration of subcutaneous tissue with NAC-enriched tumescent solution is a safe procedure. This observation justifies further research including a larger group of patients. Another potential limitation stems from the fact that the tissue samples were examined shortly after AFG harvesting. The time elapsed since the harvesting could be too short for oxidative stress to fully develop, which might have an impact on both the results of the biochemical analysis of ROS and the outcomes of qRT-PCR.

## Conclusions

While the between-group difference did not reach the threshold of statistical significance, the observation that AFG retention rate in the NAC group was lower than in the control group seems to be encouraging. This finding, as well as the results of biochemical analyses, requires further research on larger participants groups and immunocompromised animal model. If the enrichment of the tumescent solution with NAC was shown to improve the retention of the graft, this technique might become a routine adjunct method used during this type of procedures.

## Electronic supplementary material

Below is the link to the electronic supplementary material.

Results of biochemical analysis, qRT-PCR and flow cytometry
