# Supplementary material for: The Impact of N-Acetylcysteine on Autologous Fat Graft: First-in-Human Pilot Study
Source: Aesthetic Plast Surg. 2020 Mar 27;45(5):2397–405. doi: 10.1007/s00266-020-01633-1 (PMC8481185; doi:10.1007/s00266-020-01633-1)
Supplement: Supplementary file 1 — Results of biochemical analysis, qRT-PCR and flow cytometry [file 266_2020_1633_MOESM1_ESM.pdf]

Supplemental Digital Content 1.

| Patient | Group | Quantitative Real-Time Reverse Transcription Polymerase Chain Reaction | | | | | | | | | Biochemical Evaluation of Oxidative Stress | | | | | | | | Cytometric Analysis | | | |
|---|---|---|---|---|---|---|---|---|---|---|---|---|---|---|---|---|---|---|---|---|---|---|
| | | $Log_{10}$ of normalized expression | | | | | | | | | ROS (fresh) | ROS (frozen) | SOD Activity (fresh) | SOD Activity (frozen) | SOD Concentration (fresh) | SOD Concentration (frozen) | NO (fresh) | NO (frozen) | Apoptotic cells | Dead cells | Live cells | Live cell concentration |
| | | PPAR-γ | C/EBP β | VEGFA | ANGPT2 | GPX-3 | hsCAT | hsSOD | iNOS | HO-1 | p.d.u. | p.d.u. | inhibition rate % | inhibition rate % | U/ml | U/ml | μM | μM | n | n | n | n/μL |
| 1 | C | -4.17 | -0.89 | -1.76 | -2.13 | -0.67 | -0.56 | -0.66 | -4.35 | -4.56 | 1745.3 | n/a | 95.53 | n/a | 1.55 | n/a | 12.34 | n/a | n/a | n/a | n/a | n/a |
| 2 | C | -4.11 | -0.49 | -1.98 | -1.93 | -0.82 | -0.40 | -0.87 | -4.02 | -4.44 | 1484.0 | 41895.33 | 53.31 | 34.25 | 0.08 | 0.00 | 10.54 | 8.20 | 792 | 1072 | 18137 | 1893.87 |
| 3 | C | -4.21 | -0.58 | -1.93 | -1.56 | -0.84 | -0.30 | -0.74 | -4.59 | -4.12 | 1944.3 | 50438.83 | 77.34 | 53.22 | 0.66 | 0.12 | 10.16 | 10.07 | 3031 | 2941 | 13927 | 902.04 |
| 4 | C | -5.12 | -0.73 | -2.24 | -1.31 | -0.76 | -0.62 | -0.95 | -4.38 | -4.43 | 1246.0 | 25937.17 | 54.43 | 40.96 | 0.10 | 0.01 | 12.68 | 0.50 | 3552 | 6010 | 11516 | 453.80 |
| 5 | C | -4.06 | -0.59 | -1.91 | -1.45 | -0.57 | -0.29 | -0.69 | -4.05 | -3.87 | 1311.0 | 31121 | 78.55 | 66.28 | 0.70 | 0.33 | 12.24 | 7.50 | 2943 | 1048 | 16180 | 979.60 |
| 6 | C | -4.61 | -0.53 | -2.28 | -0.87 | -1.00 | -0.69 | -0.98 | -4.60 | -4.44 | 1617.7 | 15051.67 | 85.28 | 70.81 | 0.97 | 0.45 | 11.42 | 12.93 | 5596 | 6435 | 8391 | 351.29 |
| 7 | C | -3.76 | -0.50 | -2.29 | -0.37 | -0.70 | -0.66 | -1.08 | -3.96 | n/a | 1347.0 | 11105.83 | 80.37 | 70.10 | 0.76 | 0.43 | 10.19 | 4.23 | 2856 | 8910 | 9074 | 454.62 |
| 8 | C | -4.59 | -0.70 | -2.40 | -1.74 | -1.38 | -0.87 | -1.39 | -5.35 | -4.79 | 1461.3 | 26254.83 | 72.28 | 60.68 | 0.50 | 0.21 | 13.11 | 1.85 | 4545 | 5572 | 10241 | 229.95 |
| 9 | C | -4.55 | -0.81 | -2.50 | -1.07 | -0.80 | -0.65 | -1.05 | -4.09 | -4.27 | 1886.3 | 18445.5 | 48.24 | 54.06 | 0.04 | 0.09 | 12.22 | 1.84 | 2013 | 11474 | 9184 | 1131.34 |
| 10 | C | -4.66 | -0.86 | -3.00 | -1.91 | -0.97 | -0.83 | -1.01 | -4.10 | -3.94 | 1281.3 | 28177.17 | 62.67 | 56.11 | 0.24 | 0.12 | 10.70 | 0.82 | 4023 | 9002 | 9265 | 610.66 |
| 11 | C | -3.88 | -0.77 | -2.15 | -1.61 | -0.36 | 0.35 | -0.68 | -3.93 | -3.62 | 1357.7 | 17045.17 | 70.73 | 70.95 | 0.45 | 0.45 | 10.42 | 2.22 | 8659 | 7011 | 7225 | 354.05 |
| 12 | C | -3.97 | -0.67 | -1.98 | -2.09 | -0.18 | 0.19 | -0.86 | -3.61 | -4.13 | 1881.3 | 15063.67 | 87.67 | 83.96 | 1.08 | 0.91 | 12.17 | 1.40 | 5548 | 11337 | 4923 | 266.83 |
| 13 | C | -4.03 | -0.60 | -2.27 | -2.53 | -0.29 | 0.19 | -0.60 | -4.34 | -3.92 | 1906.0 | 36294.83 | 78.11 | 70.59 | 0.68 | 0.44 | 10.31 | 8.49 | 4904 | 11554 | 5767 | 413.41 |
| 14 | C | -3.73 | -0.37 | -2.13 | -1.68 | -0.44 | 0.33 | -0.60 | -3.65 | -3.56 | 2933.7 | 10331.5 | 76.01 | 71.70 | 0.61 | 0.47 | 15.90 | 1.72 | 5107 | 8845 | 7128 | 258.15 |
| 15 | C | -3.55 | -0.49 | -2.20 | -2.31 | -0.24 | 0.41 | -0.66 | -3.69 | -3.87 | 1299.7 | 14329.5 | 82.98 | 77.12 | 0.87 | 0.65 | 16.87 | 4.68 | 6946 | 9315 | 5437 | 266.30 |
| **Mean** | | -4.20 | -0.64 | -2.20 | -1.64 | -0.67 | -0.29 | -0.85 | -4.18 | -4.14 | 1646.8 | 24392.29 | 73.57 | 62.91 | 0.62 | 0.33 | 12.09 | 4.75 | 4322.50 | 7180.43 | 9742.50 | 611.85 |
| **SD** | | 0.43 | 0.15 | 0.3 | 0.57 | 0.33 | 0.46 | 0.22 | 0.45 | 0.36 | 424.0 | 11744.35 | 13.08 | 13.36 | 0.40 | 0.25 | 1.95 | 3.84 | 1964.29 | 3442.49 | 3839.36 | 452.43 |
| 1 | E | -4.59 | -0.96 | -1.84 | -2.20 | -0.67 | -0.64 | -1.16 | -4.57 | -4.94 | 1514.0 | n/a | 100.48 | n/a | 1.95 | n/a | 13.81 | n/a | n/a | n/a | n/a | n/a |
| 2 | E | -4.42 | -0.78 | -2.18 | -2.05 | -1.02 | -0.49 | -1.22 | -4.57 | -4.81 | 1328.3 | 36285.83 | 56.46 | 48.25 | 0.12 | 0.04 | 10.67 | 3.73 | 287 | 404 | 19274 | 2075.87 |
| 3 | E | -4.44 | -0.63 | -1.99 | -2.08 | -0.98 | -0.16 | -0.79 | -4.09 | -4.33 | 1982.0 | 45117.67 | 87.35 | 68.90 | 1.07 | 0.40 | 12.25 | 3.91 | 2181 | 2584 | 15242 | 767.32 |
| 4 | E | -4.13 | -0.58 | -1.97 | -1.51 | -0.46 | -0.29 | -0.71 | -4.45 | -3.90 | 1116.3 | 10716.67 | 66.78 | 63.79 | 0.34 | 0.27 | 9.72 | 0.81 | 5303 | 3653 | 11861 | 554.18 |
| 5 | E | -3.99 | -0.59 | -1.90 | -1.96 | -0.53 | -0.28 | -0.71 | -4.05 | -4.40 | 1018.7 | 33860.83 | 85.28 | 78.54 | 0.97 | 0.70 | 11.62 | 4.93 | 4327 | 2244 | 15057 | 1123.86 |
| 6 | E | -4.54 | -0.36 | -2.21 | -0.51 | -0.91 | -0.54 | -0.87 | -4.71 | -4.41 | 1464.0 | 13728.83 | 77.84 | 69.48 | 0.67 | 0.41 | 10.00 | 1.24 | 9221 | 3609 | 7663 | 176.93 |
| 7 | E | -4.73 | -0.56 | -2.10 | -0.69 | -0.83 | -0.63 | -1.14 | -5.03 | -4.31 | 1720.3 | 9567.333 | 84.47 | 77.74 | 0.93 | 0.67 | 9.47 | 1.03 | 2673 | 10308 | 8939 | 644.58 |
| 8 | E | -4.94 | -0.59 | -2.43 | -1.33 | -1.42 | -0.85 | -1.33 | -5.08 | -4.63 | 1212.3 | 11048 | 77.66 | 63.26 | 0.67 | 0.26 | 13.39 | 1.27 | 4744 | 6854 | 9369 | 157.81 |
| 9 | E | -2.92 | -0.47 | -2.98 | -1.22 | -0.90 | -0.61 | -0.89 | -4.25 | -3.90 | 2230.3 | 13315.17 | 57.07 | 61.62 | 0.13 | 0.22 | 11.84 | 1.15 | 1996 | 12028 | 8503 | 590.79 |
| 10 | E | -4.77 | -0.76 | -2.98 | -1.88 | -0.98 | -0.82 | -1.01 | -4.18 | -3.93 | 1538.3 | 26008 | 60.93 | 66.77 | 0.21 | 0.34 | 15.64 | 0.75 | 4052 | 10166 | 8300 | 553.66 |
| 11 | E | -4.23 | -0.78 | -2.13 | -1.92 | -0.37 | 0.08 | -0.70 | -4.15 | -3.92 | 1340.0 | 20718.5 | 72.91 | 73.57 | 0.51 | 0.53 | 10.38 | 1.04 | 8381 | 7257 | 7556 | 330.84 |
| 12 | E | -4.11 | -0.95 | -2.18 | -1.78 | -0.32 | 0.15 | -0.87 | -3.77 | -4.16 | 1837.7 | 28689.5 | 85.12 | 84.01 | 0.96 | 0.91 | 12.26 | 2.42 | 5838 | 9873 | 5981 | 440.39 |
| 13 | E | -3.89 | -0.57 | -2.32 | -2.19 | -0.29 | 0.25 | -0.56 | -4.08 | -3.69 | 1375.7 | 35275.5 | 81.04 | 74.19 | 0.79 | 0.55 | 10.49 | 3.93 | 2828 | 12335 | 5748 | 513.58 |
| 14 | E | -4.12 | -0.67 | -2.90 | -0.88 | -0.89 | -0.07 | -0.65 | -3.73 | -3.60 | 2124.3 | 13427.17 | 74.85 | 70.99 | 0.57 | 0.45 | 16.39 | 1.09 | 8455 | 8157 | 4741 | 175.95 |
| 15 | E | -3.84 | -0.63 | -2.12 | -2.18 | -0.34 | 0.30 | -0.59 | -4.25 | -3.53 | 939.3 | 7850 | 85.37 | 83.34 | 0.97 | 0.88 | 16.19 | 4.72 | 5751 | 10109 | 5657 | 279.80 |
| **Mean** | | -4.24 | -0.66 | -2.28 | -1.63 | -0.73 | -0.31 | -0.88 | -4.33 | -4.16 | 1516.1 | 21829.21 | 76.91 | 70.32 | 0.72 | 0.47 | 12.27 | 2.29 | 4716.93 | 7112.93 | 9563.64 | 598.97 |
| **SD** | | 0.49 | 0.16 | 0.38 | 0.57 | 0.33 | 0.39 | 0.24 | 0.4 | 0.43 | 379.8 | 11762.11 | 11.94 | 9.17 | 0.45 | 0.24 | 2.26 | 1.53 | 2565.63 | 3800.43 | 4129.14 | 481.75 |
| **P value C vs E** | | 0.173 | 0.650 | 0.363 | 0.733 | 0.112 | 0.820 | 0.955 | 0.027 | 0.925 | 0.233 | 0.158 | 0.023 | 0.003 | 0.041 | 0.004 | 0.955 | 0.009 | 0.638 | 0.875 | 0.73 | 0.73 |

ROS. reactive oxygen species; NO. nitric oxide; SOD. superoxide dismutase; C. control group; E. experimental group.
